# Arthritis sensory and motor scale: predicting functional deficits from the clinical score in collagen-induced arthritis

**DOI:** 10.1186/s13075-019-2047-z

**Published:** 2019-12-04

**Authors:** Anne-Laure Mausset-Bonnefont, Maïlys Cren, Rita Vicente, Julie Quentin, Christian Jorgensen, Florence Apparailly, Pascale Louis-Plence

**Affiliations:** IRMB, University of Montpellier, INSERM, Montpellier, France

**Keywords:** Arthritis, Sensory and motor deficits, Operator-independent quantification, Clinical scoring, Prediction scale

## Abstract

**Background:**

In the collagen-induced arthritis (CIA) mouse model, inflammation readouts are usually quantified using operator-dependent clinical scoring systems, and no systematic relationship with functional deficits has been detected. In this study, we extensively quantified sensory and motor deficits in CIA mice during natural disease progression and therapeutic treatment. Then, we used these data to build a scale to predict functional deficits on the basis of the classical clinical score.

**Methods:**

Using the CIA mouse model, we longitudinally screened multiple approaches to assess locomotion (open field test, Catwalk™), sensitivity (Von Frey, Hargreaves, static weight-bearing tests), and inflammation (skin temperature), and identified the most accurate tests to correlate sensory and motor deficits with disease severity, measured by clinical score. We then used these tests to characterize functional deficits in control (naïve and mice injected with complete Freund’s adjuvant) and CIA mice, either untreated or treated with methotrexate to prevent functional deficits. By mathematical approaches, we finally investigated the relationship between functional deficits and clinical score.

**Results:**

We found that the functional disability scores obtained with the open field, Catwalk™, Hargreaves, and skin temperature tests significantly correlated with the clinical score in CIA mice, either untreated or treated with methotrexate. Mathematical correlation showed that motor deficits, robustly characterized by two different tests, were twice more responsive than thermal sensitivity deficits.

**Conclusion:**

We propose the arthritis sensory and motor (ArthriSM) scale as a new theranostic tool to predict motor and sensory deficit based on the clinical score, in the experimental mouse model of CIA. This ArthriSM scale may facilitate the transfer of knowledge between preclinical and clinical studies.

## Background

Rheumatoid arthritis (RA) is a systemic autoimmune disease of unknown etiology that affects 1% of the adult population worldwide, making RA one of the most common chronic inflammatory diseases. RA is characterized by chronic joint inflammation, synovial hypertrophy, and progressive destruction of cartilage and bone that lead to debilitating joint pain and severe disability [1]. The clinical symptoms, such as pain and difficulty to move inflamed joints, negatively affect the patients’ well-being and ability to work, and induce psychological distress. Although numerous therapeutical approaches are currently used to alleviate symptoms, disease remission is very rare. Therefore, this pathology still needs to be better characterized in order to develop new therapies.

To this aim, animal models of RA are essential. The collagen-induced arthritis (CIA) mouse model has been extensively studied because it shares several pathological and immunological features with the human pathology, and allows testing innovative treatments in preclinical studies [2]. In the CIA model, arthritis development and severity are assessed using a clinical scoring system based on peripheral joint swelling and redness [3, 4]. Surprisingly, the relationship between clinical score and functional deficits has been scarcely studied. Although recent advances in image acquisition and analysis offer a large panel of non-invasive and automated techniques to characterize functional capacities in animals, these approaches have been rarely used to detect pain [5, 6], locomotion deficits [7–12], and bone erosion [12, 13] in different rodent models of arthritis. Moreover, no systematic and longitudinal study has been performed so far to precisely quantify sensory and motor deficits in mouse models of arthritis (under treatment or not) and to correlate them with the clinical score (i.e., the gold standard evaluation of arthritis severity).

In the present study, we performed an exhaustive quantification of the functional sensory and motor deficits in arthritic mice (CIA) as well as their prevention in animals treated with methotrexate (MTX), the main disease-modifying drug for animal models of arthritis [14, 15] and patients with RA [16]. We found a strong correlation between specific functional impairment scores (inflammation, pain, and locomotion assessment tests) and the clinical score. Therefore, we propose a new scale to predict functional deficits from the classical clinical score.

## Material and methods

### Mice

DBA/1OlaHsd male mice were purchased from Envigo Laboratories and maintained in our specific pathogen-free animal facility with 12:12 light/dark cycle, and food and water ad libitum. Arthritic mice received jellified food (Gel diet A03, Safe) in their cage. Experiments were performed in accordance with the European guidelines (directive 2010/63/EU) and were approved by the Languedoc-Roussillon Animal Research Ethics Committee (CEEA-LR no. 36), French Ministry for Higher Education and Research (project no. 01477.02), and French Health Authorities (Animal facility agreement C34-172-36).

### Collagen-induced arthritis

For these experiments, 9–10-week-old DBA/1OlaHsd male mice were randomly allocated by a blinded experimenter to different experimental groups: (i) naïve mice (no injection, *n* = 24 total); (ii) CIA control mice (vehicle), injection at the base of the tail of complete Freund’s adjuvant (CFA, Pierce) emulsified with 0.05 N acetic acid (CFA group, *n* = 15 total); (iii) untreated (*n* = 29 total) and (iv) MTX-treated CIA mice (*n* = 23 total), injection at the base of the tail of 100 μg of bovine collagen type II (bCII, MD biosciences) dissolved in 0.05 N acetic acid and emulsified in CFA complemented with 4 mg/ml H37Ra *Mycobacterium tuberculosis* (final dilution). At day 21 (D21), mice in the CFA, untreated, and MTX-treated CIA groups received an immunization booster at the base of the tail: 100 μg of bCII emulsified in incomplete Freund’s adjuvant (IFA, Pierce) for the untreated and MTX-treated CIA groups, or IFA in 0.05 N acetic acid for the CFA group. Mice in the MTX-treated CIA group were then injected with 1 mg/kg MTX (Metoject®, Nordic pharma) 3 times per week starting at D22 until the end of the experiment (D49).

**Fig. 1:**
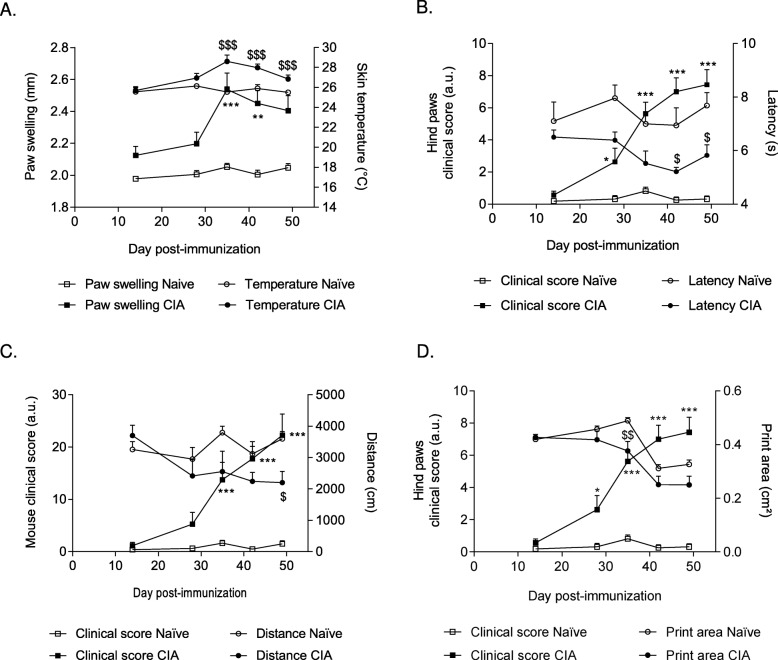
Longitudinal monitoring of inflammation, pain, and locomotor deficits in collagen-induced arthritis (CIA) mice. **a** Paw swelling (squares) and skin temperature (circles) compared in CIA mice (closed symbols) and naïve (non-immunized, open symbols) mice over time (*n* = 8). **b** Hind paw clinical score (squares) and latency before withdrawal (thermal nociception) measured with the Hargreaves test (circles) over time (*n* = 8). **c** Total clinical score (squares) and distance traveled in the open field test for 10 min (circles) (*n* = 8). **d** Hind paw clinical score (squares) and hind paw area (Catwalk™) (circles) (*n* = 8). Data were compared between CIA (closed symbols) and naïve (open symbols) mice at the same time point with the repeated two-way ANOVA test followed by Sidak’s multiple comparison test. The asterisk symbol is used for the comparison of paw swelling or clinical score between CIA and naïve mice, the dollar sign is used for the comparison of functional parameter (skin temperature, latency before withdrawal, distance traveled, or print area) between CIA and naïve mice. ^*,$^*p* < 0.05, **^,$$^*p* < 0.01, ***^,$$$^*p* < 0.001

**Fig. 2:**
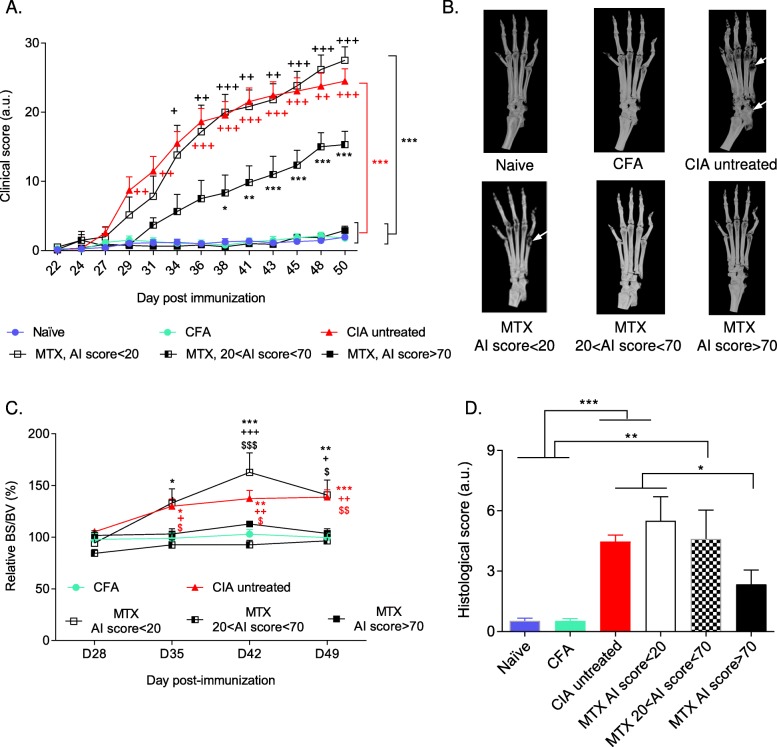
Effect of treatment with methotrexate (MTX) on clinical and functional parameters. **a** Clinical score of naïve (*n* = 16, blue circles), complete Freund’s adjuvant (CFA) (*n* = 15, green circles), untreated collagen-induced arthritis (CIA) (*n* = 21, incidence 87.5%, red triangles), and MTX-treated CIA mice subgrouped in poor responders (arthritis improvement score (AI score) < 20, *n* = 6, white squares), mild responders (20 < AI score < 70, *n* = 6, dashed squares), and high responders (AI score > 70, *n* = 11, black squares). The asterisk symbol is used for comparison with the naïve, CFA, and MTX high responder groups, and the plus sign for comparison with the MTX mild responder group. **b** Representative 3D reconstruction images of hind paws in each experimental group at D50. Note joint destruction and bone degradation (“black” areas in bone structure) only in untreated CIA and MTX poor responder mice (arrows). **c** Ankle bone degradation measured as the relative BS/BV ratio quantified in micro-CT images. The asterisk symbol is used for comparison with the CFA group, the plus sign for comparison with the MTX mild responder group, and the dollar sign for comparison with the MTX high responder group. **d** Histological score measured in naïve (*n* = 10 paws), CFA (*n* = 9), untreated CIA (*n* = 20), MTX poor responder (*n* = 6), MTX mild responder (*n* = 5), and MTX high responder mice (*n* = 9). *^,+,$^*p* < 0.05, **^,++,$$^*p* < 0.01, ***^,+++,$$$^*p* < 0.001

**Fig. 3:**
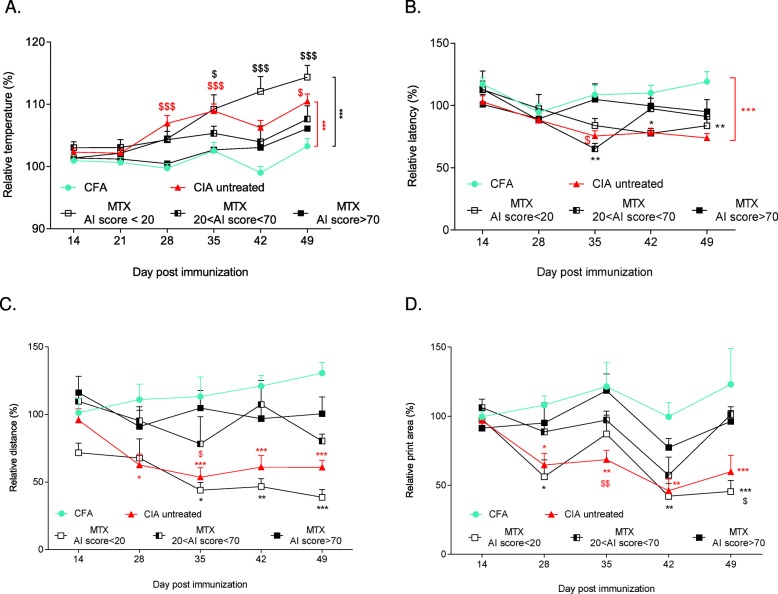
Inflammation, thermal sensitivity, and locomotion analyses in untreated and MTX-treated CIA mice. **a** Relative skin temperature in complete Freund’s adjuvant (CFA) (*n* = 28 paws, green circles), untreated collagen-induced arthritis (CIA) (*n* = 40 paws, red triangles), methotrexate (MTX) poor responder (*n* = 14 paws, white squares), MTX mild responder (*n* = 7 paws, dashed squares), and MTX high responder mice (*n* = 23 paws, black squares). **b**, Relative latency before paw withdrawal (thermal hyperalgesia) in CFA (*n* = 26 paws, green circles), untreated CIA (*n* = 40 paws, red triangles), MTX poor responder (*n* = 13 paws, white squares), MTX mild responder (*n* = 7 paws, dashed squares), and MTX high responder mice (*n* = 13 paws, black squares). **c** Relative distance traveled (open field test) by CFA (*n* = 14, green circles), untreated CIA (*n* = 20, red triangles), MTX poor responder (*n* = 6, white squares), MTX mild responder (*n* = 6, dashed squares), and MTX high responder mice (*n* = 11, black squares) in 10 min. **d** Relative print area of hind paws (Catwalk™) of CFA (*n* = 6, green circles), untreated CIA (*n* = 13, red triangles), MTX poor responder (*n* = 8, white squares), MTX mild responder (*n* = 5, dashed squares), and MTX high responder mice (*n* = 5, black squares). The asterisk symbol is used for comparison with the CFA group, and the dollar sign for comparison with the MTX high responder group; *^,$^*p* < 0.05, **^,$$^*p* < 0.01, ***^,$$$^*p* < 0.001

One experiment was performed with 2 experimental groups: naïve group (*n* = 8) and CIA group (*n* = 8, immunized mice) (cf Fig. 1). Two other independent experiments were performed with 4 experimental groups (cf Figs. 2 and 3; (1) naïve, *n* = 8 for each experiment, i.e., *n* = 16 total; (2) CFA (vehicle), *n* = 8 for each experiment but 1 animal died before the end of one of the experiments, i.e., *n* = 15 total; (3) untreated CIA, *n* = 12 for each experiment but 3 mice did not develop arthritis and were excluded from this group, i.e., *n* = 21 total; and (4) MTX-treated mice, *n* = 12 for each experiment but 1 mouse died before the end of one of the experiments, i.e., *n* = 23 total).

### Clinical score

From D21, the thickness of each hind paw was measured with a caliper (3 times per week) and arthritis severity was graded according to the previously described clinical score (maximum score of 10 per paw and of 40 per mouse) [17]. Mice with a total clinical score > 10 at D49 were considered as arthritic.

In the MTX-treated CIA group, the treatment response of each mouse was described using an arthritis improvement (AI) score that was created to mimic the American College of Rheumatology (ACR) criteria (ACR 20, 50, 70) [18] used to demonstrate the efficacy of a treatment versus a comparator in clinical studies. Accordingly, MTX-treated mice were divided in 3 subgroups in function of their AI score: (i) AI score < 20, poor responders (less than 20% improvement of the total clinical score compared with the mean clinical score obtained at D50 for the untreated CIA group), *n* = 6; (ii) 20 < AI score < 70, mild responders (20 to 70% improvement of the total clinical score), *n* = 6; and (iii) AI score > 70, high responders (more than 70% improvement of the total clinical score), *n* = 11.

### Functional tests

Functional tests (skin temperature, static weight-bearing test, Von Frey test, thermal hyperalgesia, open field, and Catwalk™ gait analysis) were performed 1 week before the immunization boost (D14) and once per week after the immunization boost for 4 consecutive weeks (D28, D35, D42, and D49).

### Skin temperature

Skin temperature was measured with an infrared thermometer (153-IRB, Bioseb, France) at 1 mm from the skin of the hind paws.

### Postural disequilibrium

To assess spontaneous pain, the weight exerted by each hind paw in static resting position was measured with a static weight-bearing test (Bioseb, France). Mice were trained to stand on their hind paws in the static weight-bearing box, with an inclined plane (65° from horizontal). Each hind paw was positioned on a separate sensor plate that independently measures the weight in grams that the animal applies on each sensor. The equipment was set to average the weight over 5 s, and at least three measurements per mouse were performed.

### Mechanical hyperalgesia (Von Frey test)

Mice were placed individually in separate plastic chambers on an elevated wire-mesh platform that allowed access to the plantar surface of each hind paw. The setup allowed testing up to 12 mice simultaneously. After an acclimation period of about 30 min, the hind paw mechanosensitivity was assessed using the automated Dynamic Plantar aesthesiometer (UgoBasile, Italy). The touch-stimulator unit was positioned directly under the hind paw mid-plantar surface. The thin steel filament was then lifted towards the paw to touch the plantar surface with increasing force. The applied force and latency time were recorded automatically at each paw withdrawal. The maximum applied force was 5 g for 30 s.

### Thermal hyperalgesia

The response to a heat stimulus was tested using a plantar test device and the method described by Hargreaves et al. (IITC Life Sciences, USA). Animals were placed in transparent Plexiglass boxes on an elevated Plexiglass floor. The setup allowed testing up to 12 mice simultaneously. They were unrestrained and acclimated to their individual boxes for approximately 30 min. The mobile infrared source was positioned under the mid-plantar surface of the hind paw. A constant intensity (37 °C), radiant heat source was applied, and the withdrawal latency was automatically recorded upon paw withdrawal from the heat source. Withdrawal was defined as paw lifting, licking, or flinching. To prevent tissue damage, the radiant source was applied for not longer than 20 s. For each animal, three measurements of each hind paw were taken with a 5-min interval between measurements and averaged to obtain a single mean value for each hind paw.

### Global locomotion

The open field test (Infrared Actimeter, Bioseb, France) is commonly used to assess locomotor, exploratory, and anxiety-like behavior. It consists of an empty and bright square arena (45 × 45 × 20 cm), surrounded by walls to prevent animals from escaping. Each animal was placed in the center of the arena, and its behavior recorded with infrared beams for 10 min. Different parameters were measured: traveled distance, speed, activity and rest time, and fast and slow movements. Traveled distance was chosen as representative of all the parameters measured.

### Gait analysis

Gait was analyzed using the Catwalk™ system (Noldus Information Technology, Netherlands) in a room where all lights were turned off except for the computer screen light that was strongly reduced. Briefly, mice were allowed to voluntarily cross a 100-cm-long, 5-cm-wide walkway with a glass platform illuminated by green fluorescent light. No habituation was needed for this experiment because mice willingly and freely crossed the walkway. When a mouse paw touches the glass floor, the green light, which enters through the long edge of the glass, is scattered, and an illuminated image is produced, in which the light intensity correlates with the force exerted by the paw. Footprints captured by a high speed camera placed under the glass floor were analyzed using the CatWalk™ XT 10.1 software. The CatWalk™ analysis software assesses many result parameters, of which we selected a few. Mice were allowed to spontaneously cross the walkway as often as needed to obtain three compliant runs, defined as runs with a minimum of three consecutive complete step cycles of all four paws without stopping or hesitation. Three to six trials were recorded, and three compliant runs (reviewed manually by the experimenter) were analyzed for each animal and each time point. Data are reported as the average of the three runs per mouse.

### Micro-CT image acquisition and analysis

Animals were anesthetized with a mixture of ketamine (100 mg/kg) and xylazine (10 mg/kg) in saline solution, and then, in vivo images were acquired with a micro-CT scanner (Skyscan 1176; Bruker, Brussels, Belgium) at 18-μm voxel size (50 kV, 200 μA, 0.5 mm aluminum filter, 360° rotation, 0.77° rotation step).

Hind limbs were stretched and placed in a polystyrene holder to image toes, foot, ankle, and distal tibiae without irradiating the abdomen. The ankle joint bones were analyzed with the 3D data analysis software CTAnalyzer (Skyscan). The regions of interest were selected relative to a growth plate reference slice. Bone analysis was performed over 1.644 mm, starting at 0.900 mm above the growth plate. The ratio of bone surface over bone volume (BS/BV), reflecting bone degradation, was calculated.

### Histopathology

Joints were collected at the end of the experiment (D50), fixed in 4% formaldehyde, and decalcified in PBS with 14% EDTA for at least 3 weeks. They were then embedded in paraffin, and sections stained with hematoxylin and eosin and safranin-O. Each ankle was scored in a blind manner by two independent operators. Histopathological features of synovial inflammation, and bone and cartilage erosion were scored as separate readout parameters with grading scores ranging from 0 (healthy, intact) to 3 (severe), leading to a maximal score of 9 for each paw.

### Data analysis and statistics

Data are presented as the mean ± SEM, and significance was determined using GraphPad Prism (GraphPad Software).

For paw swelling and clinical score, data are expressed as the mean ± SEM of individual data (Figs. 1 and 2). The clinical score was calculated either for hind paws or for the whole mouse according to the functional parameters measured. Since latency before withdrawal (thermal sensitivity) and print areas (Catwalk™) have been measured on hind paws and global distance traveled by the mice has been measured on the whole mice, each parameter was correlated to either hind paws (thermal sensitivity and print area) or global mouse (distance traveled) clinical score (Fig. 1).

For histological analysis, data are expressed as the mean ± SEM of the histological score measured for one hind paw. For functional parameters shown in Figs. 2 and 3, relative data are expressed as the mean ± SEM of the ratio between this parameter measured in each individual mouse and the mean of this parameter measured in the naïve group at the same time point. The ratio analysis for functional parameters was used in order to pool the results obtained from two independent experiments.

Functional parameters and clinical scores were compared between experimental groups and naïve one at each time point with a repeated two-way ANOVA (time and treatment effects) followed by post hoc Sidak’s (Fig. 1) or Tukey’s (Figs. 2 and 3) multiple comparison tests. A value of *p* < 0.05 was considered statistically significant. On the graphs, the significance symbol color corresponds to the group used for the comparison. Histological scores were analyzed with a parametric one-way ANOVA followed by post hoc Tukey’s multiple comparison test.

The different numbers of paws or animals between experiments and groups are explained by either (1) the death of some mice along the course of the experiments or (2) the rare inability to measure some parameters at one time point (different technical reasons). Since the statistical comparisons for functional parameters were performed by repeated two-way ANOVA, the lack of one value at a time point obliged to exclude the values corresponding to the same paw or mouse from all other time points.

For mathematical correlations, data obtained from all groups (three independent experiments) were pooled, whatever the time point. Each functional parameter was plotted in function of clinical score. The linear equations obtained were used to calculate the theoretical value of functional parameter for each clinical score. The theoretical value obtained for a null clinical score was set as the reference. We then calculated the relative functional deficit (expressed in %) for each parameter according to the formula:

Relative functional deficit = (reference − theoretical value for each clinical score)/reference × 100.

Finally, we plotted these relative functional deficits as a function of clinical score (expressed as % of the maximal score).

## Results

### Clinical score and sensory and motor deficits in the CIA mouse model

To investigate the relationship between functional disabilities and clinical score in arthritis, we first quantified inflammation, pain, and locomotor deficits in healthy (naïve, non-immunized) and arthritic (CIA group) mice, and monitored disease progression using gold standard parameters (paw swelling and clinical score).

Skin temperature measurement, as an inflammation readout, showed a bell-shaped curve, with a peak of temperature at D35 (28.61 ± 0.64 °C in CIA mice vs 25.56 ± 0.49 °C in naïve mice, *p* < 0.001) concomitantly with the peak in paw swelling (2.54 ± 0.10 mm in CIA mice compared with 2.05 ± 0.02 mm in naïve mice, *p* < 0.001) (Fig. 1a), showing that skin temperature and paw swelling behaved similarly during the disease course.

We next investigated the link between clinical score and thermal hyperalgesia. Using Hargreaves’ test, we observed that withdrawal latency was significantly reduced in CIA mice compared with naïve animals, from D42 onwards (Fig. 1b). Moreover, in CIA mice, this decreased latency was concomitant with the increase in the hind paw clinical score, suggesting an increased thermal sensitivity in arthritic animals.

These results suggest that the clinical score mirrors the objective inflammation parameters (skin temperature and thermal hyperalgesia).

Then, to assess locomotor deficits, we used two non-invasive and operator-independent locomotor tests: the open field test to quantify global locomotor activity, and the Catwalk™ system to measure dynamic gait patterns. The total clinical score of CIA mice increased significantly from D35 compared with naïve animals (*p* < 0.001). Conversely, the traveled distance (open field test) by CIA mice progressively decreased (*p* < 0.05 at D49) (Fig. 1c).

The Catwalk™ setup allows the quantification of several parameters related to gait and individual paw prints during spontaneous locomotion. Among these parameters, we selected the print area as a representative example of the results obtained. To be consistent with the results on hind paw inflammation, we present only the results for hind paws but the same effects were observed on front paws (data not shown). In CIA mice, the hind paw clinical score increased significantly from D28 (*p* < 0.05), and concomitantly, their print area during spontaneous locomotion progressively decreased compared with naïve animals (*p* < 0.01 at D35) (Fig. 1d). It is noteworthy that the run duration was not statistically different between naïve and CIA mice all along the course of the experiment (data not shown).

Overall, these results show that the disease severity, as reflected by clinical score, mirrors the sensory and motor alterations.

### Clinical score and functional deficit reduction after treatment with MTX

We then assessed functional deficits and clinical score during treatment with MTX. To this aim, we monitored the clinical score and analyzed the structural and histological alterations of joints from untreated CIA mice, MTX-treated CIA mice, and controls (naïve and CFA groups) over time. Depending on the arthritis improvement (AI) score that is based on the clinical response to MTX, we divided mice from the MTX group in three subgroups: (i) poor responders (AI score < 20), (ii) mild responders (20 < AI score < 70), and (iii) high responders (AI score > 70) (see the “Material and Methods” section). The clinical score progressively increased in untreated CIA mice and was significantly different from that of controls (naïve and CFA groups) and of the MTX high responder group, starting from D31 to D50 (experiment end) (Fig. 2a, red symbols on the right of the graph, *p* < 0.001). The clinical scores of the MTX poor responder group were similar to those of the untreated CIA group, and significantly different compared with non-arthritic controls and the MTX high-responder group from D34 onwards (Fig. 2a, black symbols on the right of the graph, *p* < 0.001). The clinical scores of the MTX mild responder group were intermediate between those of non-arthritic controls and untreated CIA mice (*p* < 0.05 at D38, *p* < 0.01 at D41, and *p* < 0.001 from D43 onward, compared with controls and MTX high responders).

At D50, bone architecture was monitored using micro-CT. As observed in representative 3D reconstructed images, bone degradation was evidenced in CIA and in MTX poor responder mice (Fig. 2b), but not in MTX mild and high responder mice and in non-arthritic controls. The relative bone surface/bone volume (BS/BV) ratio, an objective readout of bone degradation, confirmed higher ankle bone degradation in untreated CIA mice than in the CFA and MTX mild and high responder groups from D35 onwards (*p* < 0.05) (Fig. 2c). In the untreated CIA group, bone erosion increased progressively over time (D49, *p* < 0.001 compared with the CFA, and *p* < 0.01 compared with the MTX mild and high responder groups) (Fig. 2c). In MTX poor responder mice, bone degradation was comparable to that of untreated CIA mice, with significant differences compared with the CFA and MTX mild responder groups from D35 onwards.

Tissue damage was confirmed by post-mortem histological staining of ankle joints. Histological scoring of synovial inflammation, and bone and cartilage erosion was significantly higher in the untreated CIA group than in the non-arthritic controls (naïve, CFA, *p* < 0.001) and MTX high responder groups (*p* < 0.05) (Fig. 2d). The histological score of the MTX poor responder group was similar to that of untreated CIA mice and significantly higher than that of non-arthritic controls (*p* < 0.001) and MTX high responder mice (*p* < 0.05) (Fig. 2d). The histological score of the MTX mild responder group was also significantly higher than that of non-arthritic controls (*p* < 0.01, Fig. 2d).

Overall, these findings indicate that the clinical score changes in the different experimental groups faithfully mirror the histological alterations and structural joint degradation over time.

Then, analysis of the sensory and motor capacities showed features of MTX-treated mice significantly different depending on the response to MTX and therefore to the clinical score. Indeed, compared with the non-arthritic control (CFA) and MTX high responder groups, relative skin temperature was higher in the untreated CIA group from D28 (red stars on the right of the graph and red dollars, respectively, *p* < 0.001) and also in the MTX poor responder group from D35 (black symbols on the right of the graph, *p* < 0.01 at D35 and *p* < 0.001 from D42 for CFA comparison; *p* < 0.05 at D35, *p* < 0.001 at D42 and D49 for MTX high responder group comparison) (Fig. 3a). The results for MTX mild responder mice were intermediate between those of the MTX poor and high responder groups (no significant difference with these groups).

Similarly, compared with the CFA group, relative withdrawal latency measured after thermal stimulation was significantly decreased in untreated CIA mice from D35 onwards (red symbols on the right of the graph, *p* < 0.001; Fig. 3b) and also in MTX poor responder mice, but only from D42 (*p* < 0.05 at D42 and *p* < 0.01 at D49). Conversely, relative withdrawal latency in MTX mild and high responder mice was similar to that in control mice (CFA group) from D42 onwards.

The relative global locomotor activity (i.e., the distance spontaneously traveled in 10 min in the open field test) was significantly reduced in CIA and MTX poor responder mice compared with the CFA group, from D28 and D35, respectively, until the experiment end (*p* < 0.001 at D49), but not in MTX mild and high responder mice (Fig. 3c).

Finally, motor deficit analysis using the Catwalk™ system showed that the relative hind paw print area was reduced in untreated CIA and MTX poor responder mice from D28 to the experiment end (*p* < 0.001 at D49) compared with controls (CFA) (Fig. 3d), but not in MTX mild and high responder mice.

Altogether, these data demonstrate that the amplitude of sensory and motor deficits mirrors the arthritis severity and response to treatment.

### A new scale to predict functional deficits from the clinical score

**Fig. 4:**
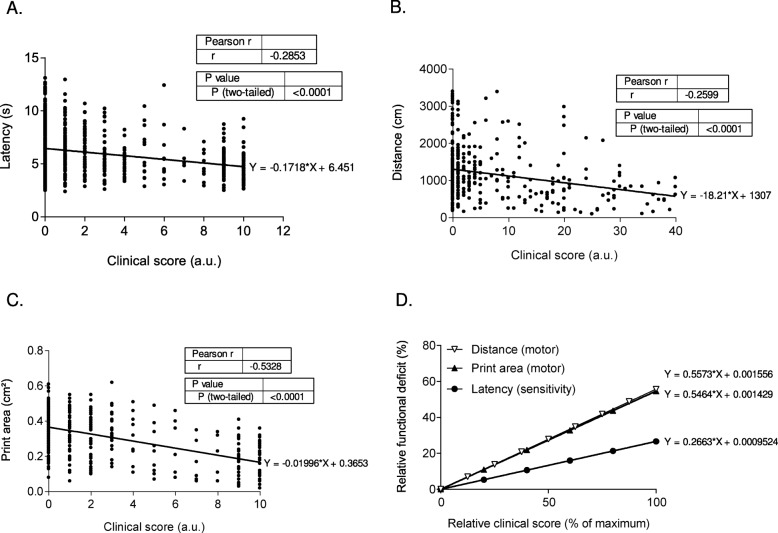
Prediction of locomotor and sensory deficits using the arthritis sensory and motor (ArthriSM) scale. **a** Correlation of the latency before paw withdrawal (thermal hyperalgesia) with the clinical score (*n* = 775 pairs). **b** Correlation of the distance traveled (global locomotion; open field test) with the clinical score (*n* = 375 pairs). **c** Correlation of the print area (gait analysis with Catwalk™) with the clinical score (*n* = 444 pairs). Linear regression equations, Spearman’s correlation coefficient *r*, and statistical significance (*p* value) are indicated in each graph. **d** ArthriSM scale. Relative functional deficits for the three assessed functional parameters are plotted according to the percentage of the maximal clinical score. The linear equations are noted for each curve. Data were pooled for all experimental groups and from three independent experiments

We then investigated whether the clinical score could be correlated with functional deficits. To this aim, we pooled all the previous data to evaluate the mathematical correlations between functional parameters and clinical score. We plotted latency before withdrawal (thermal sensitivity), distance traveled (general locomotion), and print area (gait analysis) as a function of the clinical scores (Fig. 4a–c) obtained in three independent experiments, after pooling all time points and all mouse groups. We found that all three parameters were significantly and negatively correlated with the clinical score (*r* = − 0.29, *p* < 0.0001; *r* = − 0.26, *p* < 0.0001; and *r* = − 0.53, *p* < 0.0001, respectively). We then calculated the relative functional deficit for the three parameters and plotted the linear equations of these deficits as a function of the relative total clinical score (Fig. 4d). This plot showed that motor deficits were twice more responsive to a modification of the clinical score than thermal sensitivity deficits. Moreover, the linear regression slopes for locomotor deficits quantified using the Catwalk™ (print area) and open field (distance) tests overlapped (Fig. 4d). This indicates that a given clinical score corresponds to the same relative motor deficit obtained with two completely different tests. We propose that this plot could be used as a new scale that we named arthritis sensory and motor scale, or ArthriSM scale, to predict locomotor and sensory deficits in mouse models of RA just by measuring the classical clinical score. As an example, a clinical score of 50% of the maximal score might induce a thermal sensitivity deficit of approximately 13% and a motor deficit of approximately 27–28% (see equations in Fig. 4d).

## Discussion

In the present study, we quantified inflammation, pain, and locomotor deficits in untreated and MTX-treated CIA mice and controls to objectively grade functional deficits over the disease course and in response to treatment. We found that sensory and motor deficits were correlated with the severity of arthritis and propose a new tool, the ArthriSM scale, to predict functional deficits based on the clinical score. To our knowledge, this is the first detailed correlation between clinical score and functional deficits in the CIA mouse model. Hayer et al. [12] already showed a relationship between gait parameters and the extent of cartilage and bone erosion during arthritis course, but no exhaustive study has been performed so far to correlate functional deficits with the clinical score.

The robustness of ArthriSM scale relies on the intensive and operator-independent characterization of the sensory and motor capacities of untreated and MTX-treated CIA mice compared with controls. We took advantage of the variable response to MTX to grade the prevention of functional deficits according to the clinical score. Like in previous imaging studies [12, 13], we showed that ankle bone degradation was increased in arthritic mice. Moreover, MTX poor responders exhibited the same bone degradation as untreated CIA mice, and significantly higher erosion compared with controls and also mild and MTX high responders. The histological score confirmed that joint tissues from arthritic mice (untreated CIA and MTX poor responder mice) were more inflamed/eroded than in controls, whereas tissues from MTX mild and high responder groups were protected. These results are in agreement with previous studies [19, 20] and validated our experimental approach using distinct subgroups of MTX responders.

The ArthriSM scale also relies on the discrimination between sensory and motor relevant parameters that enable to precisely quantify functional deficits. In agreement with previous reports, we found that the skin temperature changes in arthritic mice followed the same pattern as inflammation monitored by paw swelling [21]. Moreover, we showed for the first time that the temperature peak in arthritic mice (untreated and MTX poor responder mice) was abolished in MTX mild and high responder mice. In accordance with Al-Abd et al. [6], we found that thermal sensitivity, measured with the Hargreaves test, was significantly increased in arthritic mice compared with controls. Moreover, thermal hyperalgesia was reduced in MTX high responder mice compared with MTX poor and mild responders. Therefore, our results highlight the tight relationship between clinical score and pain-like readouts and justify including thermal sensitivity in the ArthriSM scale.

To evaluate the correlation between arthritis severity and locomotor disabilities, we assessed dynamic gait with Catwalk™ (print area) and global locomotion with the open field test. The hind paw print area progressively decreased in arthritic mice compared with controls, as well as the maximal paw pressure intensity, stance time, and stride length (data not shown). This reduction was inversely correlated with the clinical score increase and was not due to a variation of speed duration (data not shown). The decrease in print area was previously observed in human TNF transgenic mice, a chronic inflammatory erosive model of RA [12], and in mice and rats after intra-articular injection of LPS [8] or carrageenan [10], but not the longitudinal correlation between gait parameters and clinical score severity. On the other hand, Vincelette et al. [7] found that the paw print area increased with disease progression, possibly due to the device used in their study: a treadmill coupled to a videographic system that might force animals to stabilize their paw contacts. Conversely, the Catwalk™ system allows mice to move freely on the glass floor. We also found that the print area in MTX mild and high responders and in MTX poor responder mice was similar to that of controls and untreated CIA mice, respectively. These results demonstrate that gait analysis is well correlated with arthritis severity, and corroborate the study by Simjee et al. [22] showing that gait abnormalities in rats with adjuvant-induced arthritis were reversed by treatment with MTX. We finally demonstrated that spontaneous exploratory locomotion (open field test) was decreased in arthritic mice compared with controls and was inversely correlated with the clinical score. This corroborates previous studies showing a significant decrease in free locomotion in the open field arena [6, 23, 24] and the correlation between its reduction and arthritis severity [25]. Global locomotion in MTX mild and high responder mice was similar to that of CFA controls. This is in agreement with the study by Hartog et al. [25] showing that MTX treatment from D21 onwards increases by 25% the global locomotor activity in treated compared with untreated CIA mice. Altogether, our results show that locomotion is tightly related to the clinical score in both treated and untreated CIA mice.

Among the large battery of tests we used, we found that the Von Frey test (mechanical sensitivity) was inappropriate for mice with severe arthritis because they could not place their hind paws properly on the metal grid platform (data not shown). Only one previous study used this test to assess mechanical sensitivity in arthritic mice and only up to D38 [5]. Similarly, the static weight-bearing test did not evidence any difference in hind limb pressure intensity in untreated CIA mice (data not shown) because they often had bilateral arthritis. This test would be more suitable for models of unilateral arthritis, for instance after unilateral injection of CFA [9, 26], carrageenan [10], or LPS [8].

Altogether, our results show that sensory and motor function alterations during arthritis progression and upon treatment with a disease-modifying drug mirror the clinical score changes. This prompted us to perform mathematical correlations between functional parameters and clinical score. We then used the linear regression equations to create the ArthriSM scale that plots relative functional deficits in function of the relative clinical score. With this original scale, we demonstrated that for any given clinical score, (i) sensory function is less affected than the motor function, and that (ii) independent and distinct locomotor testing setups quantify the same functional deficit (similar slopes). This last finding highlights the robustness of the ArthriSM scale to predict sensory and motor deficits based on the clinical score.

In our study, we used the CIA model on DBA/1 mice since (1) it shares several pathological and immunological features with human RA pathology [2] and (2) DBA/1 mice are genetically susceptible to develop arthritis [27]. Moreover, we chose to use male mice since the majority of studies published on CIA models used male gender and it is also known that the major difference between CIA in mice and RA in humans is the lack of sex bias in the animal model [28]. However, it would be interesting to investigate the correlation between clinical score and functional phenotyping in other RA models, mice strains, mice gender, and/or upon other therapeutic treatment, in order to generalize the ArthriSM scale to any RA models.

## Conclusions

To our knowledge, this is the first study that evidences a strong correlation between functional deficits and clinical scoring in CIA mouse model. The next step would be to further investigate and validate this correlation on other mice strains, gender, therapeutic treatments, and arthritis models. In the clinic, rheumatologists use the DAS28 scoring system that combines pain assessment in 28 joints, biological parameters, and subjective evaluation of RA activity by the patient (for review [29]) to obtain a precise and global score for each patient. In preclinical models, researchers routinely quantify the arthritis progression or regression using semi-quantitative clinical scoring systems. With the ArthriSM scale, we propose a new way to assess disease activity by predicting functional deficits based on the clinical score. Should this scale be validated on different arthritis models, we suggest that it could be combined with clinical score and biological parameters, like for the DAS28 system, to establish a unique global score that might help to assess disease progression or regression and propose animal models that better mimic the human pathology.

## Data Availability

The datasets used and/or analyzed during the current study are available from the corresponding author on reasonable request.

## Abbreviations

ACR: American College of Rheumatology
AI score: Arthritis improvement score
ArthriSM scale: Arthritis sensory and motor scale
BS/BV: Bone surface/bone volume ratio
CFA: Complete Freund’s adjuvant
CIA: Collagen-induced arthritis
DAS: Disease activity score
EDTA: Éthylènediaminetétraacétique
IFA: Incomplete Freund’s adjuvant
LPS: Lipopolysaccharide
MTX: Methotrexate
PBS: Phosphate buffer saline
RA: Rheumatoid arthritis
TNF: Tumor necrosis factor
